# Corrosion of Carbon Steel by *Shewanella chilikensis* DC57 Under Thiosulphate and Nitrate Reducing Conditions

**DOI:** 10.3389/fbioe.2022.825776

**Published:** 2022-03-10

**Authors:** Silvia J. Salgar-Chaparro, Johanna Tarazona, Laura L. Machuca

**Affiliations:** Curtin Corrosion Centre, WA School of Mines, Minerals, Energy and Chemical Engineering, Curtin University, Perth, WA, Australia

**Keywords:** microbiologically influenced corrosion, carbon steel, localised corrosion, biofilm, electron acceptor

## Abstract

*Shewanella chilikensis* DC57 is a bacterial strain isolated from a corrosion failure in a floating oil production system. Previous studies have indicated that this microorganism has potential to trigger corrosion of carbon steel through several metabolic pathways identified in its genome. In this study we evaluated the corrosion of carbon steel by *S. chilikensis* in the presence of thiosulphate or nitrate as terminal electron acceptors of the anaerobic respiration. Electrochemical response of carbon steel to the biofilm formation revealed differences in the corrosion process under the different electron acceptors conditions. Microscopic examination of the metal surface confirmed that *S. chilikensis* induced corrosion in both scenarios; however, in the presence of thiosulfate *S. chilikensis* triggered a higher pitting corrosion rate, whereas in presence of nitrate it promoted higher uniform corrosion. This study demonstrates the importance of understanding the metabolic versatility of microbes in order to assess the MIC risk of industrial facilities.

## Introduction

Corrosion is the deterioration of metals by an electrochemical reaction with the environment (Speight 2014). Under aerobic conditions, oxygen is the most common reactant in the corrosion reaction of metals, whereas, under anaerobic conditions, microbiologically influenced corrosion (MIC) is believed to be the major cause of corrosion related failures (Iino et al., 2015a). MIC results from the formation of biofilm communities on the metal surface, which can produce corrosive metabolites and extracellular polymers, resulting in changes on the metal/solution interface and the corrosion behaviour (Moura et al., 2013). Costs related to corrosion prevention, mitigation, and repair were estimated to be around $2.9 trillion dollars for 2018 (Little et al., 2020).

MIC frequently causes uniform and localised corrosion of metals through a variety of mechanisms (Miller et al., 2018), ranging from the direct microbial metabolism of metal (extracellular electron transfer EET-MIC) to enhanced metal deterioration due to microbiologically induced changes in fluid chemistry (chemical-MIC) (Enning and Garrelfs 2014; Dou et al., 2019). Causative microorganisms of the MIC phenomenon in anaerobic environments include organisms with different metabolic capabilities such as sulphate reduction, fermentation, iron reduction, nitrate reduction and methanogenesis (Dinh et al., 2004; Marciales et al., 2019; Palacios et al., 2019). Although the implications of all these microbial groups in MIC has been widely demonstrated, the estimation of the MIC risk in industrial assets has rarely ever considered the presence and activity of all of these. Reason for this is that despite the dramatic increase in the number of studies during the last three decades (Hashemi et al., 2017), there are still remarkable knowledge gaps in the understanding of factors that trigger the MIC phenomenon (Little et al., 2020).

One of the complexities of MIC assessment is that microorganisms are metabolically versatile (Selim et al., 2017), some species more than others, which possess challenges in evaluating the effect of nutrients and other environmental factors on the MIC risk. For example, species from the genus *Shewanella* possess a broad respiratory versatility and use an array of organic and inorganic compounds that include nitrate, nitrite, thiosulphate, elemental sulphur, iron (III), manganese (III), fumarate, even insoluble metal oxides when oxygen is not available (Beliaev and Saffarini 1998; Ruebush et al., 2006;Burns and Dichristina, 2009; Miller et al., 2018). Thus, *Shewanella* can cause chemical-MIC from the release of metabolic products, such as nitrite and hydrogen sulphide, and can trigger EET-MIC when harvest energy from metals (Wang et al., 2020). Therefore, versatile microorganisms can colonize a variety of oxic and anoxic environments. This metabolic versatility makes it difficult to understand their potential contribution to the corrosion process and fit it within a risk categorization.

To develop a better understanding of the contribution of versatile microorganisms in biofilms formed on industrial assets, the corrosion behaviour of *Shewanella chilikensis* DC57 was assessed under nitrate and thiosulfate reducing conditions*. S. chilikensis* is a bacterium isolated from corroded structures in a floating oil production system in Australia (Salgar-Chaparro et al., 2020a). The corrosive effect on carbon steel by this microorganism was demonstrated in a separate study where both nitrate and thiosulphate were present in the test solution (Salgar-Chaparro et al., 2020b). However, the key metabolic pathway involved in the severe pitting corrosion observed in the study could not be identified. In this investigation, electrochemical and surface analysis were carried out to assess pitting corrosion by *S. chilikensis* under the different electron acceptors.

## Materials and Methods

### Sample Preparation

AISI 1030 carbon steel was used as test material for the MIC evaluation. Round coupons with an exposed area of 1.27 cm^2^ were prepared as follows: an electrical connection was established *via* a copper wire soldered to the coupon’s surface. Subsequently, coupons were electro-coated with a protective epoxy (Powercron 6000CX, PPG Industrial coatings) and wire was removed except from the samples used for electrochemical measurements. Later, one side of the coupons was wet ground sequentially up to grid 600 by SiC-paper and used as a working surface. The polished specimens were washed with Milli-Q water, degreased with acetone, washed with ethanol, and dried with nitrogen gas. Before immersion, the coupons were weighed and sterilised by 15 min of ultraviolet (UV) radiation.

### Corrosion Experiments

Corrosion experiments were conducted in anaerobic CDC Biofilm Reactors (Biosurface Technologies Corporation). A total of 4 reactors (abiotic and biotic) were setup, two for each condition. Supplemented artificial seawater with the following composition was used as test solution: 20 g/L of sea salts (Sigma), 20 mM of Na-lactate, 20 mM Na-acetate, 9 mM Na-pyruvate, 9 mM ammonium chloride, and 4 mM Na-bicarbonate. Additionally, 10 mM of sodium nitrate or 10 mM of sodium thiosulphate were added to the reactors depending on the condition of testing. The pH of the test solution was adjusted to 7.3 ± 0.2 with 1N NaOH. Anaerobic conditions were maintained by continuous sparging of the test solution with filter sterilised nitrogen gas at a flow rate of 30 ml/min. The baffle within the reactor was set to 50 rpm to maintain a homogeneous solution throughout the test. Biotic reactors were inoculated with an active culture of *S. chilikensis* DC57 in a concentration of 2.0×10^6^ cells/mL. After an initial 72 h of operation under batch condition, in which no additional nutrients were added to the reactors, continuous flow of fresh test solution was initiated at a rate of 4.37 ml/h which replaced 30% of the test solution daily.

### Electrochemical Evaluation

Electrochemical measurements were conducted using a conventional three-electrode cell assembly. A double junction Ag/AgCl electrode (SSC) was used as reference electrode, a graphite rod was used as counter electrode, and two electrically connected coupons were used as working electrodes. All electrochemical measurements were conducted using a VMP3 multichannel potentiostat (Biologic). The open circuit potential (OCP), linear polarisation resistance (LPR), and electrochemical impedance spectroscopy (EIS) were monitored daily at each condition. LPR was scanned at a rate of 0.167 mV/s in the range of −10 mV to +10 mV vs. OCP. EIS measurements were obtained in the frequency range from 10^5^ to 10^−2^ Hz with a 10 mV amplitude. Potentiodynamic polarization measurements were conducted at the last day of immersion (16th day) with a scan rate of 0.100 mV/s between −250 mV and +250 mV vs. the OCP.

### Surface Analysis

Corrosion measurements based on mass loss and pitting were conducted after 16 days of metal immersion. For this, three metal samples from each reactor were removed and cleaned using Clarke’s solution, as described in the ASTM G1 standard (ASTM, 2017). General corrosion was estimated from the metal mass loss and pitting corrosion rate was calculated from the deepest pit found in each condition, using the equation described in NACE SP-0775 standard practice (NACE, 2013). Optical measurements and coupons’ inspection to evaluate pitting corrosion were conducted in an infinite focus microscope and Alicona IFM software. A *t*-test was implemented to assess if there were statistically significant differences in the corrosion rates between coupons exposed to biotic conditions under different electron acceptors, and between coupons exposed to *S. chilikensis* and the sterile controls. All statistical analyses were performed with PAST version 4.04 (Hammer et al., 2001), and results were considered significant with *p*-value ≤ 0.05.

### Biofilm Imaging

The distribution of live and dead cells within biofilms was studied using confocal laser scanning microscopy (CLSM). Coupons were gently rinsed with sterile anaerobic PBS and stained using the FilmTracer Live/Dead biofilm viability kit (Invitrogen) following the manufacturer’s instructions. Before imaging with a Nikon A1+ confocal microscope, coupons were rinsed with sterile deionized water to remove the excess of dyes. Images were captured with a ×20 dry objective lens. The z-stacked images were captured in separate tracks using 489.3 and 561 nm lasers paired with 500–550 nm and 570–620 nm emission filters respectively.

Biofilm morphology was examined under a TESCAN CLARA field emission scanning electron microscope (FESEM). Before imaging, biofilms were fixed with the following protocol: coupons were immersed for 22 h in a PBS solution containing 2.5% glutaraldehyde and 0.15% alcian blue. Then, coupons were rinsed with PBS and dehydrated trough ethanol series (30, 50, 70, 80, 90, 95, and 100% v/v ethanol-water) for 10 min each. Dehydrated coupons were dried under nitrogen flow for 2 days, coated with a platinum layer (10 nm thick), and stored in a vacuum desiccator until imaging. Biofilms were visualized at an emission voltage of 5 kV, aperture of 30 µm and working distance of 4 mm. Energy-dispersive X-ray spectroscopy (EDS) was used to determine the elemental composition. Aztec^®^ 4.0 software (Oxford Instruments NanoAnalysis) was used for data analysis.

### Microbial Concentration and Activity

After 16 days of immersion, three coupons were collected and rinsed with sterile phosphate-buffered saline (PBS) to remove all planktonic cells. Subsequently, coupons were immersed in 10 ml of PBS and sonicated for 2 min to detach all sessile cells from the surface. Sonication was performed in cycles of 15s ON followed by 20s OFF. Finally, coupons were vortexed at maximum speed for 10s and the cell suspension was used to quantify cell numbers and microbial activity.

The numbers of viable sessile cells were determined by the culture dependent most probable number (MPN) method using the following culture medium: 20 g/L of sea salts (Sigma), 20 mM of Na-lactate, 20 mM Na-acetate, 10 mM glucose, 1.3 g/L mM casamino acids (BD), 10 mM ammonium nitrate, 10 mM Na-thiosulphate, 0.007 mM iron (II) chloride tetrahydrate, 0.007 mM manganese (II) chloride tetrahydrate, and 4 mM Na-bicarbonate. For this, 1 ml of the cell suspension was inoculated into 9 ml of culture media, and then 10-fold serial dilutions up to 10^10^ were performed in triplicate for the MPN estimation. Serial dilution vials were incubated at 40°C for 28 days, and positive growth was determined by visual inspection of changes in the turbidity and the colour of the culture media.

Adenosine triphosphate (ATP) was determined by luminescence after reaction with luciferin-luciferase using the Quench-Gone Organic Modified (QGO–M™) test kit (Luminultra Technologies Ltd.) ATP was extracted from 5 ml of the cell suspension following the kit’s manufacturer instructions. ATP measurements were collected using the PhotonMaster™ Luminometer (Luminultra Technologies Ltd.), and ATP content was calculated from the measured luminescence by comparing it with a standard.

## Results

### Corrosion Measurements

#### Uniform Corrosion by Electrochemical Measurements

**FIGURE 1:**
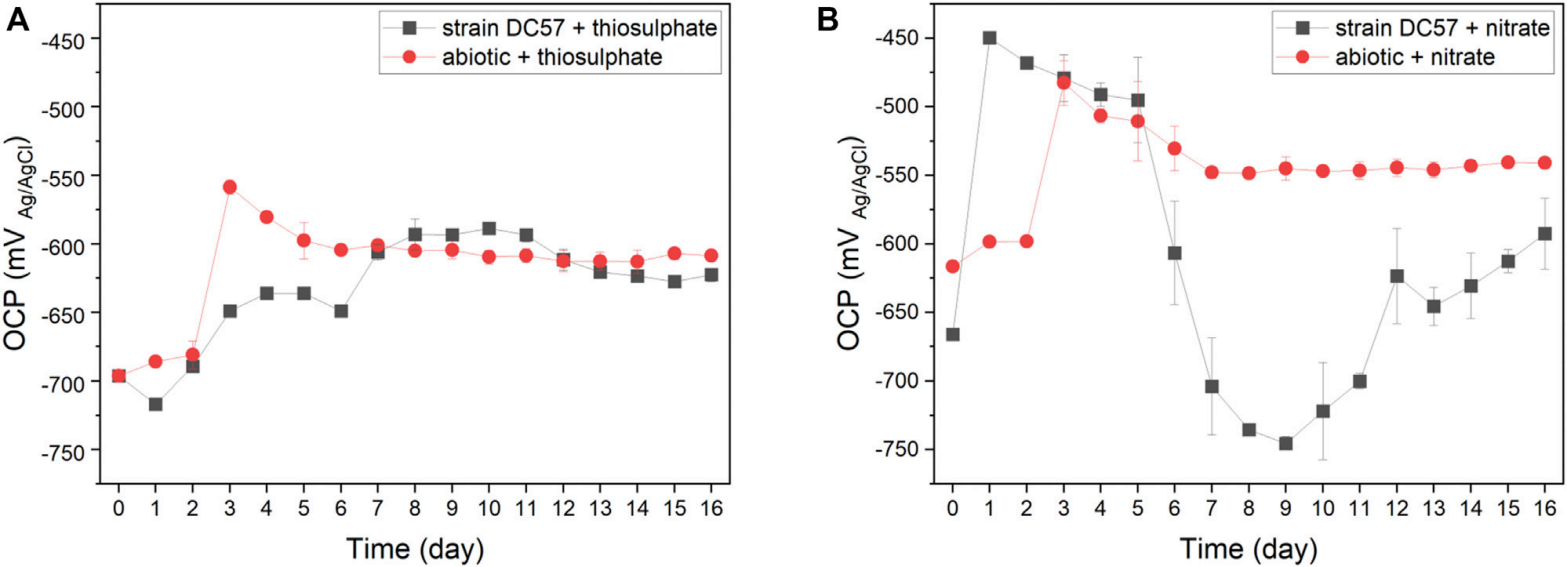
Variation of OCP vs. time during 16-days exposure to thiosulphate **(A)** and nitrate **(B)** reducing conditions.

The fluctuation of the OCP of carbon steel coupons over the immersion period is given in Figure 1. A similar pattern was observed in coupons exposed to the abiotic conditions. The OCP shifted towards more positive values, particularly after the initiation of the continuous flow of nutrients. Changes in the OCP can be attributed to variations in the metal/solution interface, such as the precipitation of salts or the formation of corrosion products layers that change the corrosion potential of the metal (Hiromoto 2019; Mansoori et al., 2019). Differently, the OCP of coupons exposed to the biotic reactors did not follow the same pattern in both conditions. In the presence of thiosulphate and *S. chilikensis* (Figure 1A), the OCP of the carbon steel shifted to more positive values, which was comparable to the abiotic control. However, in the presence of nitrate and *S. chilikensis* (Figure 1B), the OCP initially shifted to more positive values, but from day 5 to day 9, the OCP shifted towards more negative values, and after day 9 OCP shifted again to less negative values. Fluctuations of the OCP in biotic reactors suggest that biofilm formation of *S. chilikensis* resulted in changes in anodic or cathodic reactions and the acceleration of the iron dissolution as a result of the metabolic activities carried out by the bacteria.

**FIGURE 2:**
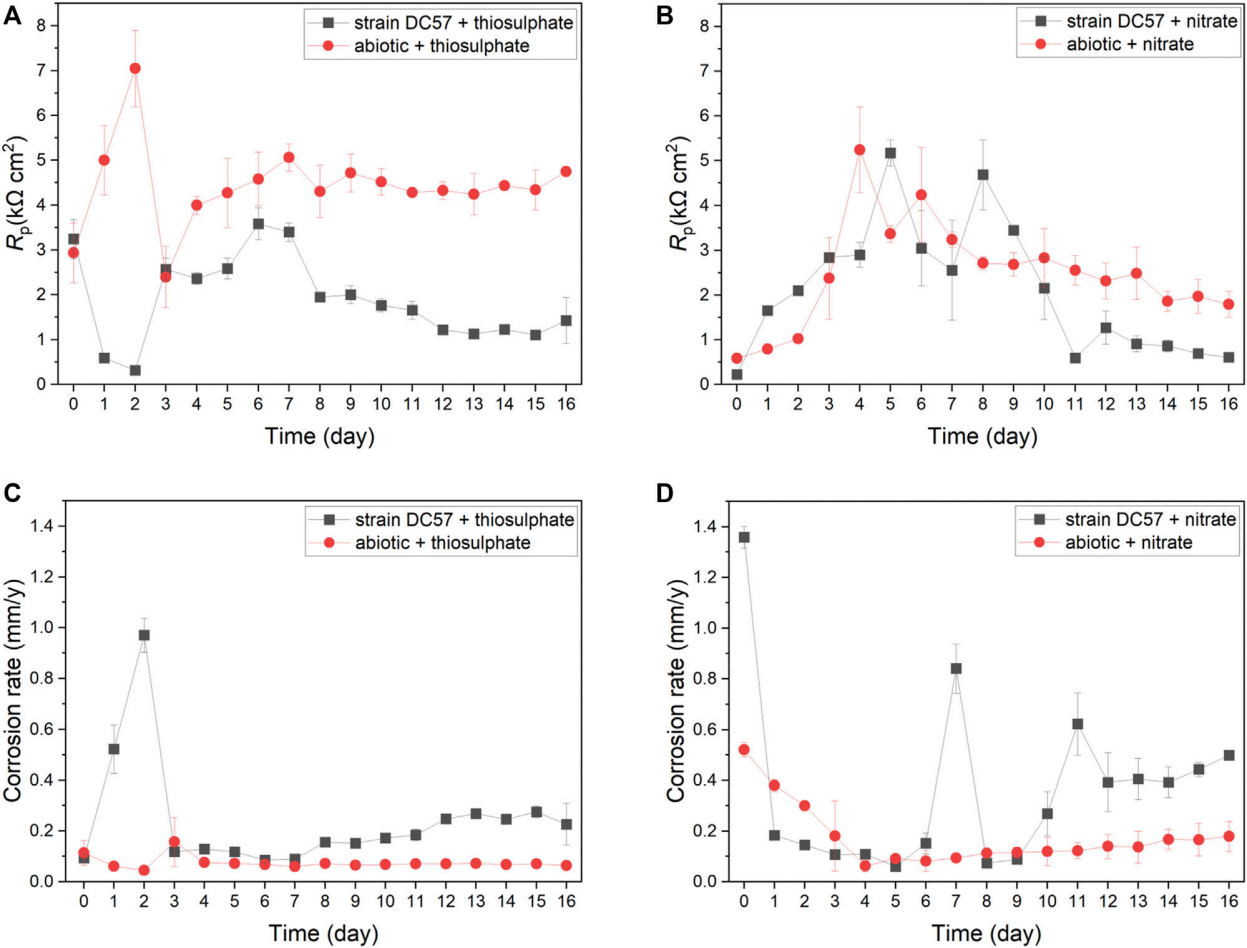
Variation of *R*p **(A,B)** and corrosion rate **(C,D)** vs. time during 16-days exposure to thiosulphate **(A,C)** and nitrate **(B,D)** reducing conditions.

Polarisation resistance (*Rp*) from LPR scans describes the transient corrosion kinetics of carbon steel coupons during the 16-days immersion. *Rp* values and corrosion rates as a function of time are shown in Figure 2. Clear differences on LPR measurements in presence of *S. chilikensis* can be found as compared to the abiotic controls. Lower *Rp* values in biotic reactors indicate higher corrosion rates (Scully 2000; Claisse 2016). In the thiosulphate condition, corrosion rate of the abiotic control was more or less stable during the 16-days immersion, displaying only a minor increase on day 3 after the initiation of the continuous flow of nutrients to the reactors. Differently, in presence of *S. chilikensis*, higher corrosion rates were observed over the batch period, and a marked reduction was seen after the initiation of the continuous flow. On the other hand, in the nitrate reducing condition, both abiotic and biotic reactors showed a reduction in the corrosion rate during the batch period, but, after initiation of the continuous flow, corrosion rate in the abiotic reactor remained stable, whereas in the presence of S*. chilikensis,* a significant fluctuation was observed. Overall, the highest corrosion rates were observed in the nitrate reducing condition compared to the thiosulphate reducing condition.

**FIGURE 3:**
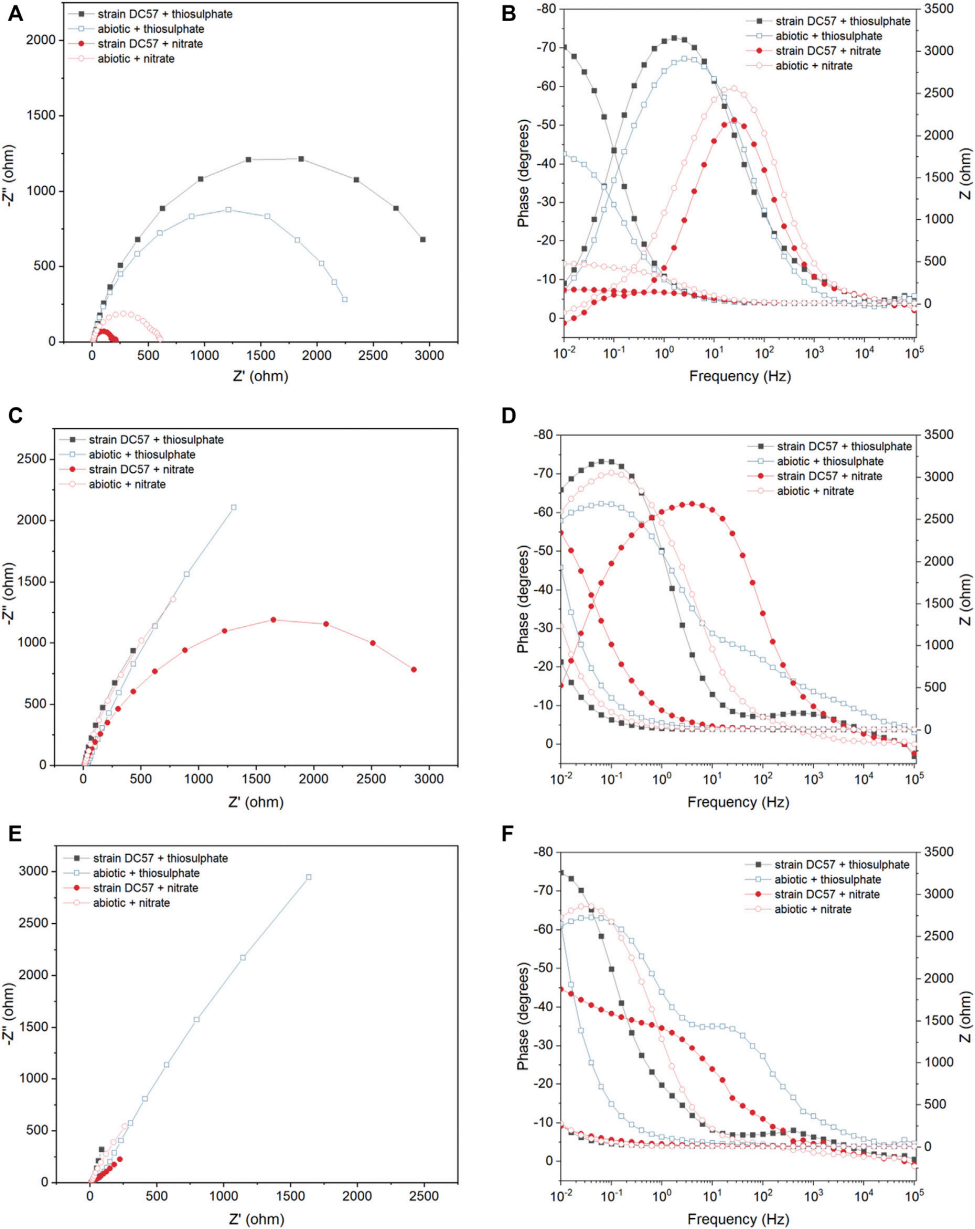
EIS Nyquist plots **(A,C,E)** and Bode plots **(B,D,F)** of carbon steel after 1 (A,B), 8 (C,D) and 16 (E,F) days exposure to thiosulphate and nitrate reducing conditions.

Figure 3 displays the Nyquist and Bode plots of carbon steel coupons exposed for 1, 8, and 16 days to the different conditions. It is clear that the impedance response of the metal changed over the test duration. A larger semicircle diameter in Nyquist plots generally indicates higher electrical resistance at the metal/solution interface, which results in lower corrosion rates (Liu et al., 2017; Cai et al., 2021). In the Nyquist plots recorded on day 1, a clear difference in the diameter of the Nyquist plots was observed between the conditions. Coupons exposed to the thiosulphate reducing condition exhibited a larger semicircle compared to the coupons exposed to the nitrate reducing condition, which suggest that coupons in presence of nitrate are more susceptible to corrosion. Nyquist plots changed during the exposure time in both abiotic and biotic reactors indicating changes at the metal/solution interface, most probably as a result of the formation of corrosion product layers and biofilm.

To get a quantitative measure of the electrochemical parameters at the metal/solution interface, the impedance spectra on Figure 3 were fitted using equivalent circuits with one time constant and two time constant (Supplementary Figure S1). The capacitors in the circuit model were not ideal capacitors, therefore, constant phase elements (CPEs) were used instead. The quality of the fitting was judged by χ2, all the χ2 values were less than 0.005 indicating a good fit of the EIS data. In the equivalent circuit model, which has been used in previous reports (Miranda et al., 2013; Cai et al., 2021), *R*_*s*_ corresponds to solution resistance, *R*_*b*_ and *CPE*_*1*_ represents the resistance and capacitance of the biofilm and/or adsorption of corrosion products on the metal surface, and *R*_*ct*_ and *CPE*_*2*_ corresponds to the charge transfer resistance and double-layer capacitance. Table 1 shows the EIS fitting results.

**TABLE 1:** Electrochemical parameters obtained from fitting the EIS spectra in Figure 3 and calculated corrosion rate.

Condition	Reactor	Day	Rs (Ω cm^2^)	Q_1-T_	Q_1-p_	Rb (Ω cm^2^)	Q_2-T_	Q_2-p_	Rct (Ω cm^2^)	CR (mm y^−1^)
Thiosulphate	Biotic	1	10.2 ± 1.2	0.0008 ± 0.0003	0.8 ± 0.04	-	-	-	2,945 ± 391	0.08 ± 0.01
		8	5.7 ± 0.3	0.0022 ± 0.0002	0.6 ± 0.04	4.0 ± 0.7	0.011 ± 0.001	0.9 ± 0.01	4,656 ± 1,039	0.05 ± 0.01
		16	11.4 ± 6.5	0.0411 ± 0.0049	3.0 ± 1.54	17.8 ± 0.7	0.066 ± 0.002	0.9 ± 0.08	1,820 ± 391	0.14 ± 0.03
	Abiotic	1	9.6 ± 0.1	0.0006 ± 0.0001	0.8 ± 0.00	-	-	-	2,725 ± 1,258	0.09 ± 0.04
		8	7.3 ± 0.2	0.0025 ± 0.0016	0.5 ± 0.08	22.4 ± 5.5	0.004 ± 0.000	0.8 ± 0.00	10,061 ± 121	0.02 ± 0.00
		16	9.2 ± 2.9	0.0015 ± 0.0002	0.6 ± 0.00	39.2 ± 22.3	0.003 ± 0.000	0.8 ± 0.04	12,604 ± 7,652	0.02 ± 0.01
Nitrate	Biotic	1	9.0 ± 0.7	0.0004 ± 0.0000	0.8 ± 0.00	—	—	—	165 ± 18	1.45 ± 0.16
		8	8.2 ± 1.7	0.0001 ± 0.0000	1.0 ± 0.00	1.7 ± 0.4	0.001 ± 0.000	0.7 ± 0.02	2,948 ± 106	0.08 ± 0.00
		16	8.7 ± 0.9	0.0165 ± 0.0026	0.4 ± 0.01	70.8 ± 45.2	0.037 ± 0.015	0.7 ± 0.11	1,692 ± 439	0.14 ± 0.03
	Abiotic	1	8.3 ± 0.2	0.0003 ± 0.0000	0.8 ± 0.00	—	—	—	438 ± 12	0.54 ± 0.01
		8	7.6 ± 0.4	0.0216 ± 0.0007	0.5 ± 0.01	2.3 ± 0.1	0.011 ± 0.007	0.8 ± 0.00	3,883 ± 1,680	0.06 ± 0.02
		16	7.0 ± 0.2	0.0220 ± 0.0019	0.5 ± 0.12	2.2 ± 0.4	0.032 ± 0.000	0.8 ± 0.00	1,828 ± 109	0.13 ± 0.00

Lower *R*_*ct*_ values observed under nitrate reducing conditions in both biotic and abiotic reactors since day 1 indicates that the presence of nitrate in the test solution leads to a more corrosive environment, which correlates with LPR measurements. Likewise, lower *R*_*ct*_ values were measured in biotic reactors compared to abiotic reactors in both conditions, indicating that biofilm formation by strain *S. chilikensis* produced an increase in corrosion rates of the carbon steel.

**FIGURE 4:**
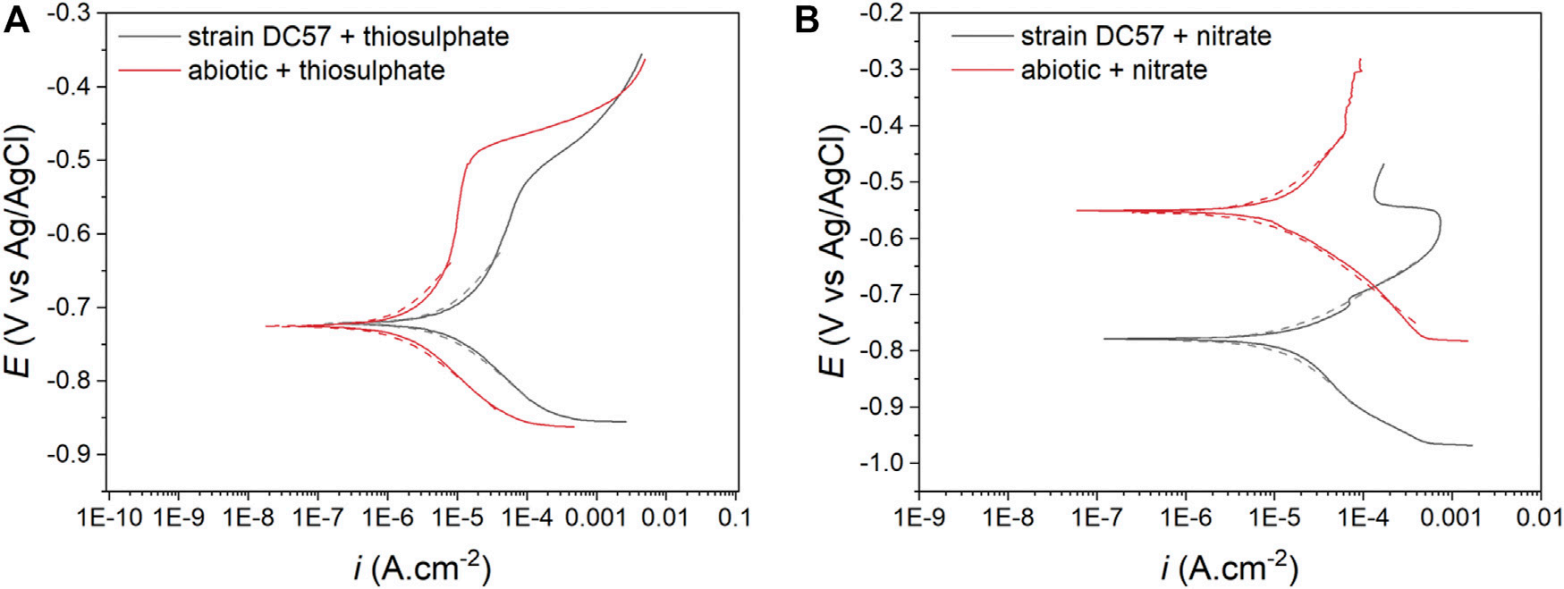
Potentiodynamic polarization curves after 16-days exposure to thiosulphate **(A)** and nitrate **(B)** reducing conditions, dashed lines represent the Tafel fitting.

The potentiodynamic polarization curves obtained after the 16-days exposure to the different conditions are shown in Figure 4. The values of anodic (*β*_a_) and cathodic (*β*_c_) Tafel slopes, corrosion potential (*E*_Corr_), corrosion current density (*i*_Corr_), and the consequent corrosion rates (CR) obtained from the polarization curves are listed in Table 2. Samples exposed to nitrate reducing conditions showed a considerable higher corrosion current density (15.8 µA/cm^2^) when compared to the samples exposed to the thiosulphate reducing conditions (7.4 µA/cm^2^). These results are consistent with other electrochemical measurements (LPR and EIS) corroborating that nitrate represents a more corrosive scenario for carbon steel. Nonetheless, greater current densities were recorded in the coupons exposed to *S. chilikensis* under thiosulfate reducing condition compared with its sterile control which ratifies that *S. chilikensis* can promote corrosion of carbon steel under both conditions at a different rate. Significant differences in current density between the biotic and abiotic reactors in the nitrate reducing condition were not recorded with the polarisation studies. It is important to highlight that potentiodynamic polarization analysis was only conducted on the last day of exposure due to the damage it can cause to the metal; differences between the sterile control and biotic conditions in the nitrate reducing conditions could have occur earlier in the test.

**TABLE 2:** Electrochemical parameters obtained from Tafel analysis.

Condition	Reactor	*β*_a_ mV/dec	*β*_c_ mV/dec	*E*_*corr*_ mV vs.Ag/AgCl	*i*_*corr*_ (µA/cm^2^)	CR (mm y^−1^)
Thiosulphate	Biotic	123.3 ± 2.5	87.6 ± 0.2	−724.2 ± 3.4	7.4 ± 0.5	0.067 ± 0.004
	Abiotic	116.2 ± 8.4	80.9 ± 4.5	−721.6 ± 5.4	1.7 ± 0.2	0.015 ± 0.001
Nitrate	Biotic	95.2 ± 2.4	139.1 ± 28.9	−741.5 ± 55.2	15.8 ± 1.6	0.145 ± 0.015
	Abiotic	195.5 ± 10.7	137.3 ± 13.6	−550.9 ± 4.0	15.4 ± 6.0	0.140 ± 0.055

#### Corrosion Rates by Mass Loss and Surface Analysis

**FIGURE 5:**
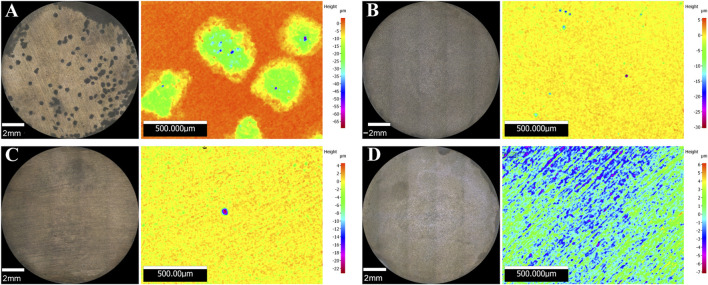
3D optical microscope surface images of coupons exposed to *S. chilikensis* under thiosulphate reducing conditions **(A)** and nitrate reducing conditions **(B)**. **(C,D)** correspond to their respective sterile controls.

**FIGURE 6:**
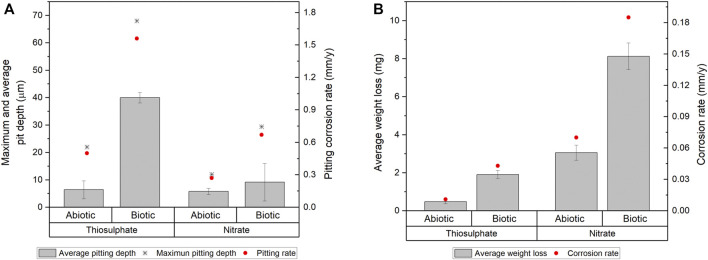
Localised corrosion **(A)** and uniform corrosion measurements **(B)** of carbon steel coupons after 16-days immersion to thiosulphate and nitrate reducing conditions.

Surface examination was conducted after the 16-days exposure to assess localised corrosion. 3D optical microscopy revealed the presence of pits in the metal surface of coupons exposed to biotic conditions (Figure 5). Although both biotic conditions exhibited localised corrosion, a notorious difference in the pit density and pit depth was observed between conditions. Higher number of pits were detected in coupons exposed to *S. chilikensis* under thiosulphate reducing conditions (Figure 5A) compared to the coupons exposed to nitrate reducing conditions (Figure 5B). Average pit depth, calculated from the ten deepest pits found in three coupons of each reactor, maximum pit depths, and pitting rates are presented in Figure 6A. Deeper pits were visualised in the presence of *S. chilikensis,* however, differences in the pit depth vs. sterile controls were only significant in the thiosulphate reducing condition (*p* ≤ 0.05; Supplementary Table S1). Differences in the pit depth between biotic conditions were also statistically significant (*p* ≤ 0.05; Supplementary Table S1), *S. chilikensis* induced deeper pits under thiosulphate reducing condition (40 ± 10 µm) compared with pits induced under nitrate reducing condition (7 ± 2 µm)*.*

Coupon’s mass loss was determined to calculate uniform corrosion rates, mass loss and average corrosion rates are presented in Figure 6B. Uniform corrosion measurements from surface analysis correlate well with the results from electrochemical measurements. Higher corrosion rates were calculated in coupons exposed to *S. chilikensis;* differences in corrosion rates between coupons exposed to biotic conditions compared to sterile controls were significant (*p* ≤ 0.05; Supplementary Table S2). Differences in the uniform corrosion rates between biotic conditions were also statistically significant (*p* ≤ 0.05; Supplementary Table S2), *S. chilikensis* induced higher uniform corrosion under nitrate reducing conditions (0.19 ± 0.01 mm/y) compared with uniform corrosion rates induced under thiosulphate reducing condition (0.04 ± 0.004 mm/y)*.*

In order to determine the impact of *S. chilikensis* in the corrosion phenomenon, corrosion rates obtained in sterile controls were subtracted from the biotic corrosion rates. According to the qualitative categorization of carbon steel corrosion rates as per NACE standard practice SP0775 (NACE, 2013), *S. chilikensis* promoted severe localised corrosion and moderate uniform corrosion under thiosulphate reducing conditions, and low localised corrosion and high uniform corrosion under nitrate reducing conditions.

### Microbiological Analyses

#### Biofilm Imaging

**FIGURE 7:**
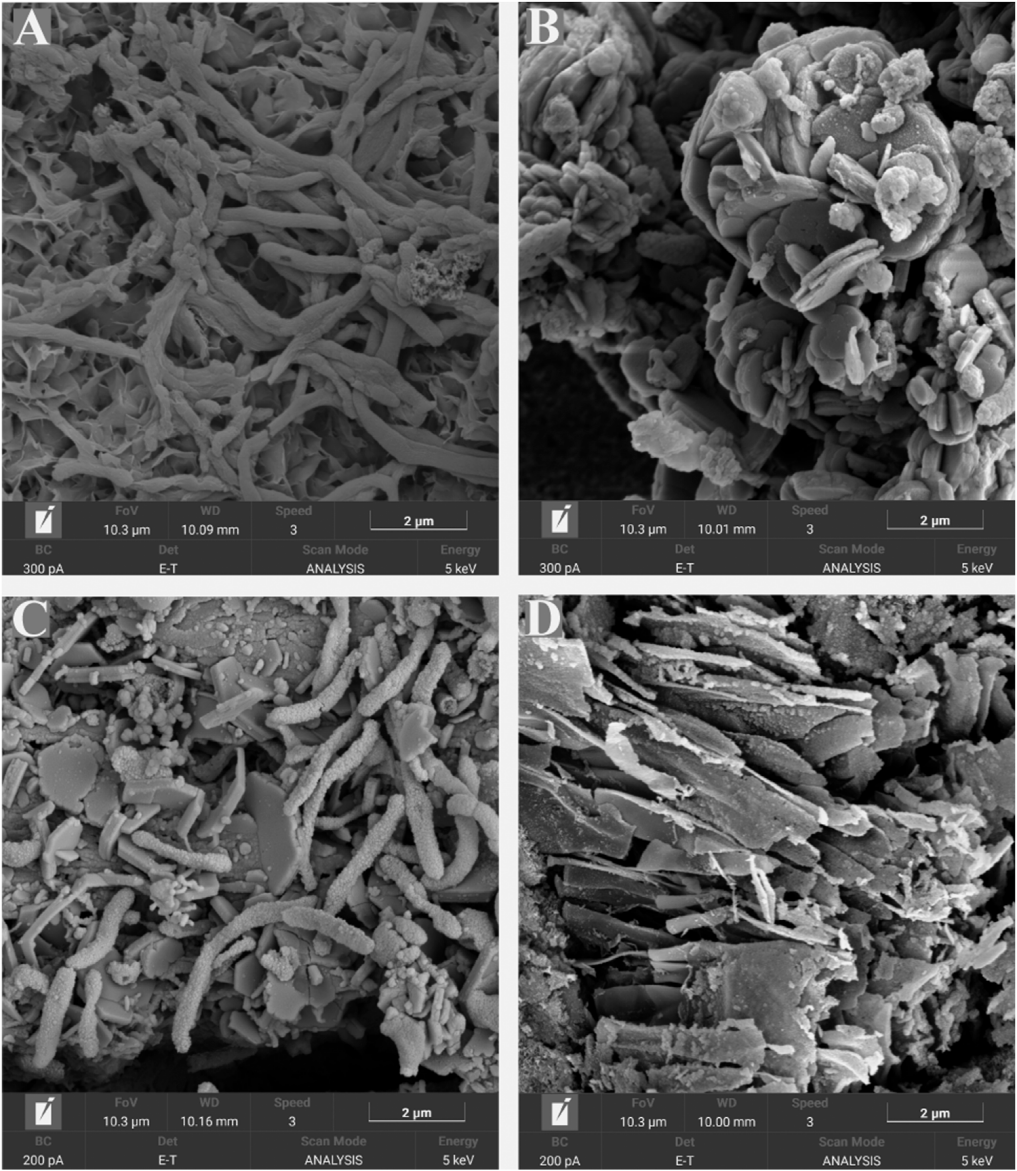
SEM micrographs of coupon’s surfaces after 16-days exposure to *S. chilikensis* under thiosulphate reducing conditions **(A)** and nitrate reducing conditions **(C)**. **(B,D)** correspond to their respective sterile controls.

FESEM micrographs of the metal surfaces are shown in Figure 7. Microscopy analysis confirmed the presence of cells attached to the surface of the coupons exposed to the biotic conditions. In the presence of *S. chilikensis,* a more complex film was observed. Different layers with corrosion products, bacteria, and extracellular polymeric substances (EPS) were noticed (Figures 7A−C). Contrarily, coupons exposed to the abiotic conditions only presented a thin layer of deposits probably resulting from salt precipitation or from abiotic corrosion reactions (Figures 7B−D).

**FIGURE 8:**
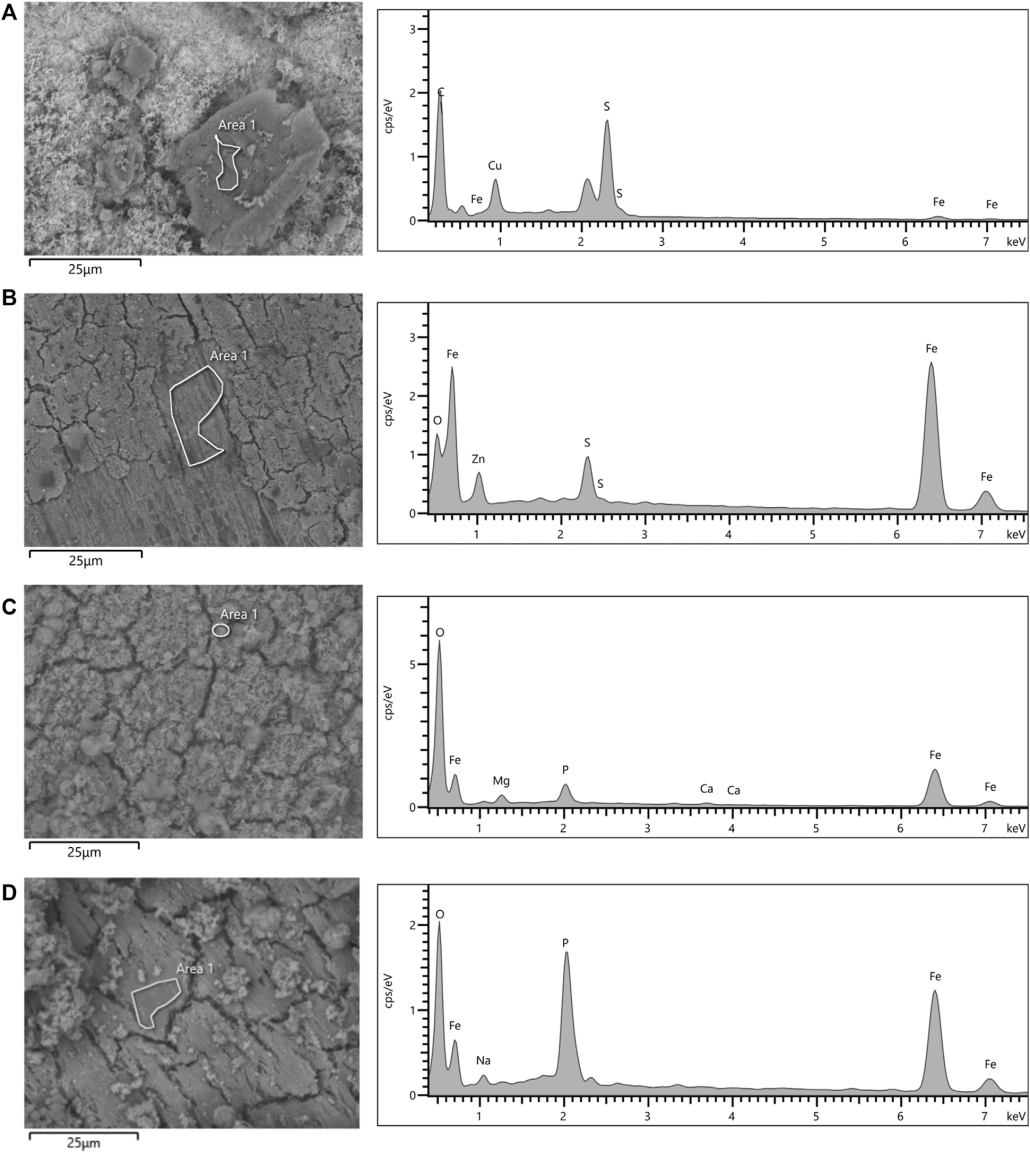
SEM micrographs and EDS spectra of coupon’s surfaces after 16-days exposure to *S. chilikensis* **(A,C)** and sterile controls **(B,D)** to thiosulphate **(A,B)** and nitrate **(C,D)** reducing conditions.

The morphology observations and elemental analysis of corrosion products is shown in Figure 8. EDS analysis indicated that corrosion products in the thiosulphate reducing condition are predominantly composed by iron and sulphur (Figures 8A,B), whereas corrosion products in the nitrate reducing condition were mainly composed by iron, oxygen, and phosphorus (Figures 8C,D). The presence of iron and sulphur in the presence of thiosulphate confirms the formation of biologically generated sulphides in the corrosion products.

**FIGURE 9:**
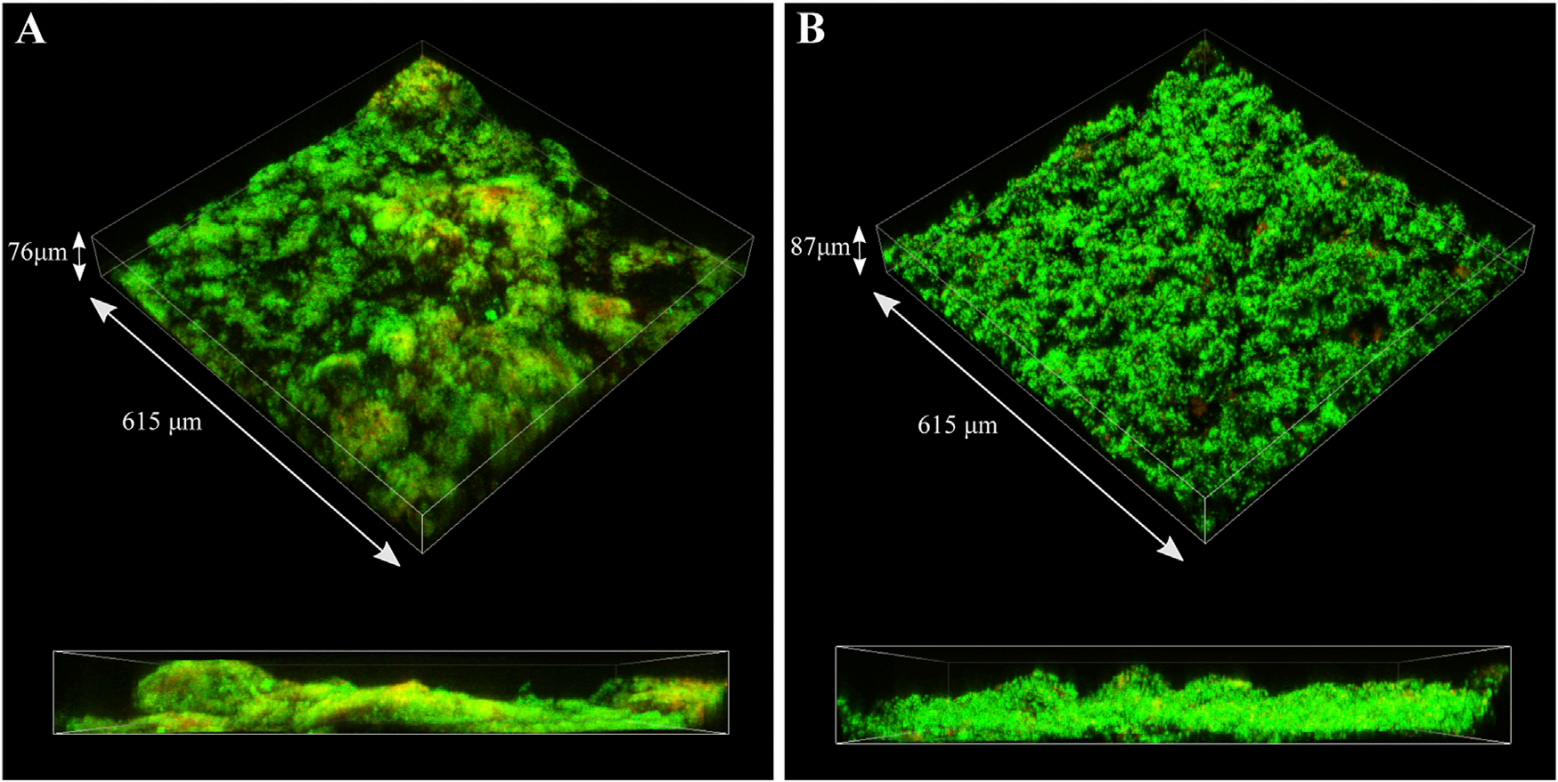
CLSM images of biofilms formed by *S. chilikensis* after 16-days exposure to thiosulphate **(A)** and nitrate **(B)** reducing conditions.

CLSM was used to identify live (green) and dead (red) cells in the biofilm matrix (Figure 9). Images revealed that *S. chilikensis* was able to form dense biofilms under both conditions. As shown in Figure 9, after 16-days exposure, cells were predominantly alive with minor concentration of cells that stained red. CLSM analysis also showed patchy surface areas without biofilm, which can generate different local environments across the metal surface.

#### Microbial Concentration and Activity

**FIGURE 10:**
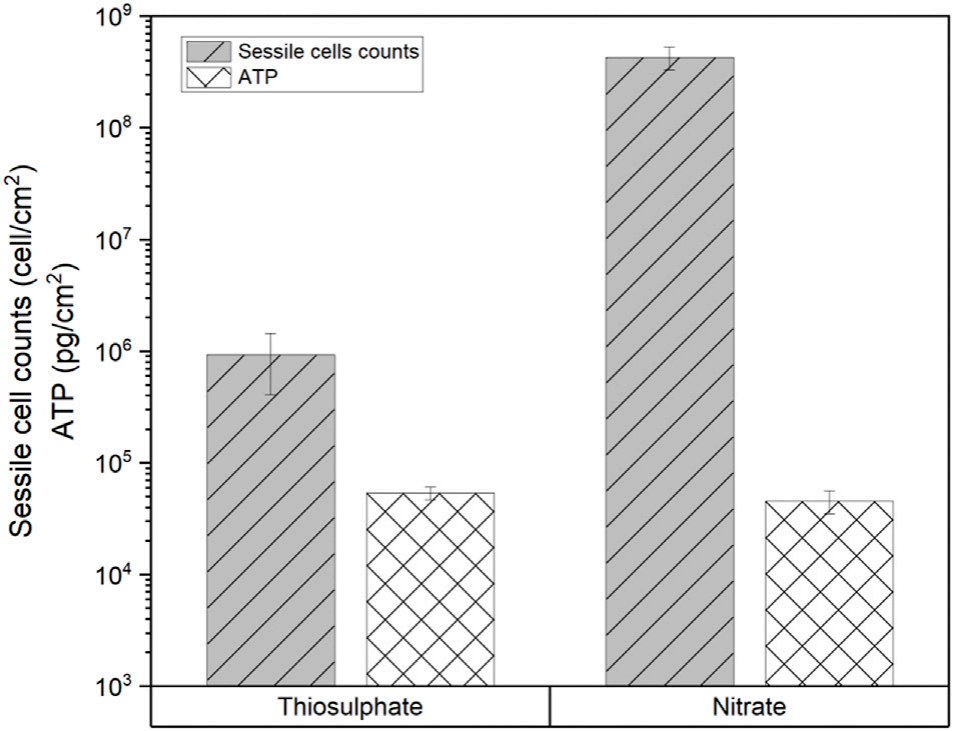
Sessile cell counts and ATP level in biofilms formed by *S. chilikensis* after 16-days exposure to thiosulphate and nitrate reducing conditions.

MPN analysis showed that biofilms in the nitrate reducing condition had 3 orders of magnitude more cells (4 × 10^8^ cells/cm^2^) than biofilms formed under thiosulphate reducing conditions (7 × 10^5^ cells/cm^2^). Interestingly, even though biofilms formed under thiosulphate reducing condition displayed lower concentration of cells compared to the biofilms formed under nitrate reducing conditions, the ATP values were slightly higher (53,772 pg/cm^2^) than in the nitrate condition (45,812 pg/cm^2^) (Figure 10), suggesting that metabolism of *S. chilikensis* using thiosulphate produced higher yield of ATP.

## Discussion

This study assessed the corrosive potential of *S. chilikensis* strain DC57 in the presence of different electron acceptors. For this, carbon steel coupons were exposed to *S. chilikensis* in presence of thiosulphate or nitrate which are chemical compounds typically found in oil productions systems. For example, nitrate injection has been extensively used by oil and gas producing companies in order to suppress reservoir souring generated by sulphate reducing bacteria (SRB) (Xu et al., 2013). Nitrate addition promotes the growth of nitrate reducing bacteria (NRB) that out-compete SRB for source of carbon and energy (Stott 2012; Iino et al., 2015a; Etique et al., 2018). Although nitrate treatment has been shown to be effective in controlling reservoir souring, it has been reported that it can pose a risk for the integrity of the downstream assets (Iino et al., 2015b; Yuk et al., 2020). For instance, corrosion promoted by NRB could take part or be accelerated if concentration of nitrate in the environment increases. MIC mechanisms supported under nitrate reducing conditions include the production of nitrite, which is an intermediate in dissimilatory nitrate reduction. Nitrite is electro-active and redox-active molecule that can oxidise the Fe^(0)^ in the steel or react with ferrous iron from the corrosion process (Liu et al., 2021a). Nitrates can also support the direct microbial metabolism of Fe^(0)^ or the consumption of cathodic H_2_ enhancing the EET-MIC mechanism (Iino et al., 2015b; Jia et al., 2017), as well as through the formation of thick biofilms that induce localized cathodic and anodic regions on the steel (Liu et al., 2021b).

On the other hand, thiosulphate has been found to be present in oilfield environments as a result of biological or chemical oxidation of sulphides. Thiosulphate can be produced by oxidation of H_2_S when oxygen is introduced in the system during processing or by the action of sulphide oxidizing organisms (Amend et al., 2004). Likewise, thiosulfate can be reduced to elemental sulphur or hydrogen sulphide by the action of the thiosulphate reducing bacteria (TRB). It has been stated that the biological turnover of thiosulfate can be rapid and potentially a more significant contributor to the sulphide concentration in oil reservoirs than the sulphides produced by sulphate reduction (Liang et al., 2014). Therefore, the presence of thiosulfate in the production systems increases the risk of MIC (Lan et al., 2012). Corrosion rates prompted by TRB have been found to be similar to the corrosion rates induced by SRB (Liang et al., 2014; Machuca et al., 2017). These two metabolic routes can accelerate corrosion through galvanic effects induced by the precipitation of iron sulphide on the steel surface (Etique et al., 2018).

Results of this investigation demonstrated that *S. chilikensis* can cause MIC under both thiosulphate and nitrate reducing conditions. However, marked differences were observed in the corrosion mechanism between the two studied scenarios. Electrochemical, weight loss and surface analyses of the metal samples after 16-days exposure showed that uniform and pitting corrosion were triggered by the bacterium. Uniform corrosion implies the oxidation of the metal across its surface by a dissolved oxidant resulting in metal mass loss, whereas, pitting corrosion is a localized form of corrosion by which pits or holes are produced in the metal (Wang et al., 2016). The weight loss data showed that the presence of *S. chilikensis* in the test solution caused 3 to 4 times more uniform corrosion than the sterile controls. The highest uniform corrosion was obtained when *S. chilikensis* was present in the nitrate reducing condition, which was 4 times greater than the uniform corrosion in the thiosulphate reducing condition. Electrochemical measurements were consistent with these results, LPR and EIS data showed that corrosion rates in coupons exposed to the *S. chilikensis* under nitrate reducing condition were greater than in the thiosulphate reducing condition. Uniform corrosion by other species of the genus *Shewanella* (*S. oneidensis* MR-1) has been previously reported when nitrate was available in the test solution (Miller et al., 2018). The authors stated that nitrite accumulation as a result of nitrate reduction prompted general corrosion of the metal surface. As mentioned before, nitrites can cause corrosion by interacting directly with the elemental iron from the steel or by reacting with the ferrous iron from the proton reduction reaction (Weber et al., 2006). Moreover, the heterogeneous distribution of the biofilm may have generated nitrite concentration cells, resulting in the localised corrosion observed in this condition. Most common corrosion products formed from nitrite corrosion of mild steel are maghemite (Fe_2_O_3_), goethite (FeOOH), lepidocrocite (FeOOH) and magnetite (Fe_3_O_4_) (Lee, Kim, and Kim 2012), which correlates well with the elements detected in the EDS analysis of coupons exposed to nitrate reducing condition (Fe and O), although, further analyses are required to identify which corrosion products are formed by the action of *S. chilikensis* under nitrate reducing conditions.

Profilometer analysis instead revealed that *S. chilikensis* induced a greater localised corrosion when thiosulphate was present in the test solution. Under thiosulphate reducing conditions *S. chilikensis* produces hydrogen sulphide, which is widely known by its corrosive effect on mild steel (Duret-Thual 2014; Zheng, 2015; Jia et al., 2018). Indeed, corrosion of mild steel by thiosulfate reducing bacteria has normally been related to very active pitting corrosion with penetrations up to 10 mm per year (Campaignolle et al., 1996; Magot et al., 1997). It has been reported that under specific conditions, thiosulfate alone can cause pitting corrosion (Newman et al., 1989; Zhang, Carcea, and Newman 2015). Nevertheless, absence of localised damage in sterile controls proved that the localized attack was only stimulated by the metabolic activities of *S. chilikensis.* Although *S. chilikensis* caused MIC under both conditions, the severe localised corrosion observed under thiosulphate reducing conditions represent a higher MIC risk for industrial infrastructure compared to the uniform corrosion observed under nitrate reducing conditions. Pitting corrosion can lead to the rapid perforation of a local area, resulting in structural failures (Fu et al., 2021). Therefore, when assessing the risk of MIC, it is very important to consider the specific metabolic reactions that microorganisms can perform according to the environmental conditions.

## Conclusion

Results of this investigation have demonstrated that the extent of microbiologically influenced corrosion is highly dependent on the characteristics of the environment. In this study, *S. chilikensis* DC57 a bacterium previously isolated from an oilfield corrosion failure, promoted severe pitting corrosion of carbon steel when thiosulphate was used as electron acceptor. Under nitrate reducing conditions, *S. chilikensis* increased uniform corrosion but only minor pitting corrosion was observed, despite the high concentration of cells present in the biofilm. The above reinforces the importance of the chemical and microbiological data integration to assess the potential risk of MIC in industrial systems. Therefore, evaluation of the MIC risk must consider both the microbial species present in a system but most importantly, the environmental conditions, in particular nutrient availability, and associated metabolic activities that microorganisms can perform on metallic surfaces under such conditions.

## Data Availability

The raw data supporting the conclusions of this article will be made available by the authors, without undue reservation.

## References

[B1] Amend J. P. Edwards K. J. Lyons T. W. (2004). Sulfur Biogeochemistry: Past and Present. Boulder, CO: Geological Society of America.

[B2] ASTM 2017. ASTM G1 (2017). Standard Practice for Preparing, Cleaning, and Evaluating Corrosion Test Specimens. ASTM International.

[B3] Beliaev A. S. Saffarini D. A. (1998). *Shewanella Putrefaciens mtrB* Encodes an Outer Membrane Protein Required for Fe(III) and Mn(IV) Reduction. J. Bacteriol. 180 (23), 6292–6297. 10.1128/jb.180.23.6292-6297.1998 9829939 PMC107715

[B4] Burns J. L. Dichristina T. J. (2009). Anaerobic Respiration of Elemental Sulfur and Thiosulfate by *Shewanella Oneidensis* MR-1 Requires *psrA*, a Homolog of the *phsA* Gene of *Salmonella enterica* Serovar Typhimurium LT2. Appl. Environ. Microbiol., 75(16). 10.1128/AEM.00888-09 PMC2725451 19542325

[B5] Cai D. Wu J. Chai K. (2021). Microbiologically Influenced Corrosion Behavior of Carbon Steel in the Presence of marine Bacteria *Pseudomonas Sp*. And *Vibrio Sp* . ACS Omega 6 (5), 3780–3790. 10.1021/acsomega.0c05402 33585757 PMC7876863

[B6] Campaignolle X. Caumette P. Dabosi F. Crolet J. L. (1996). “The Role of Thiosulfate on the Microbially Induced Pitting of Carbon Steel,” in CORROSION 96 (Denver: Colorado).

[B7] Claisse P. A. (2016). “Corrosion,” in Civil Engineering Materials. Editor Claisse P. A. (Boston: Butterworth-Heinemann), 339–359. 10.1016/b978-0-08-100275-9.00031-0

[B8] Dinh H. T. Kuever J. Mussmann M. Hassel A. W. Stratmann M. Widdel F. (2004). Iron Corrosion by Novel Anaerobic Microorganisms. Nature 427 (6977), 829–832. 10.1038/nature02321 14985759

[B9] Dou W. Liu J. Cai W. Wang D. Jia R. Chen S. (2019). Electrochemical Investigation of Increased Carbon Steel Corrosion via Extracellular Electron Transfer by a Sulfate Reducing Bacterium under Carbon Source Starvation. Corrosion Sci. 150, 258–267. 10.1016/j.corsci.2019.02.005

[B10] Duret-Thual C. (2014). “The Effect of H2S on the Corrosion of Steels,” in Understanding Biocorrosion. Editors Liengen T. Féron D. Basséguy R. Beech I. B. (Oxford: Woodhead Publishing), 385–407. 10.1533/9781782421252.3.385

[B11] Enning D. Garrelfs J. (2014). Corrosion of Iron by Sulfate-Reducing Bacteria: New Views of an Old Problem. Appl. Environ. Microbiol. 80 (4), 1226–1236. 10.1128/AEM.02848-13 24317078 PMC3911074

[B12] Etique M. Romaine A. Bihannic I. Gley R. Carteret C. Abdelmoula M. (2018). Abiotically or Microbially Mediated Transformations of Magnetite by Sulphide Species: The Unforeseen Role of Nitrate-Reducing Bacteria. Corrosion Sci. 142, 31–44. 10.1016/j.corsci.2018.06.032

[B13] Fu Q. Xu J. Wei B. Qin Q. Gao L. Bai Y. (2021). The Effect of Nitrate Reducing Bacteria on the Corrosion Behavior of X80 Pipeline Steel in the Soil Extract Solution of Shenyang. Int. J. Press. Vessels Piping 190, 104313. 10.1016/j.ijpvp.2021.104313

[B14] Hammer O. Harper D. Ryan P. (2001). PAST: Paleontological Statistics Software Package for Education and Data Analysis. Palaeontol. Electron. 4, 1–9.

[B15] Hashemi S. J. Bak N. Khan F. Hawboldt K. Lefsrud L. Wolodko J. (2017). Bibliometric Analysis of Microbiologically Influenced Corrosion (MIC) of Oil and Gas Engineering Systems. Corrosion 74 (4), 468–486. 10.5006/2620

[B16] Hiromoto S. (2019). “Corrosion of Metallic Biomaterials,” in Metals for Biomedical Devices. Editor Niinomi M.. Second Edition (San Diego, CA: Woodhead Publishing), 131–152. 10.1016/b978-0-08-102666-3.00004-3

[B17] Iino T. Sakamoto M. Ohkuma M. (2015a). Prolixibacter Denitrificans Sp. nov., an Iron-Corroding, Facultatively Aerobic, Nitrate-Reducing Bacterium Isolated from Crude Oil, and Emended Descriptions of the Genus Prolixibacter and Prolixibacter Bellariivorans. Int. J. Syst. Evol. Microbiol. 65 (9), 2865–2869. 10.1099/ijs.0.000343 25991662

[B18] Iino T. Ito K. Wakai S. Tsurumaru H. Ohkuma M. Harayama S. (2015b). Iron Corrosion Induced by Nonhydrogenotrophic Nitrate-Reducing *Prolixibacter* Sp. Strain MIC1-1. Appl. Environ. Microbiol. 81 (5), 1839–1846. 10.1128/AEM.03741-14 25548048 PMC4325155

[B19] Jia R. Tan J. L. Jin P. Blackwood D. J. Xu D. Gu T. (2018). Effects of Biogenic H2S on the Microbiologically Influenced Corrosion of C1018 Carbon Steel by Sulfate Reducing Desulfovibrio Vulgaris Biofilm. Corrosion Sci. 130, 1–11. 10.1016/j.corsci.2017.10.023

[B20] Jia R. Yang D. Xu D. Gu T. (2017). Electron Transfer Mediators Accelerated the Microbiologically Influence Corrosion against Carbon Steel by Nitrate Reducing *Pseudomonas aeruginosa* Biofilm. Bioelectrochemistry 118, 38–46. 10.1016/j.bioelechem.2017.06.013 28715664

[B21] Lan G. Hong H. Qing D. Wu Y. Tan H. (2012). Identification and Bio-Corrosion Behavior of *Thermoanaerobacter* CF1, a Thiosulfate Reducing Bacterium Isolated from Dagang Oil Field. Afr. J. Microbiol. Res. 6 (12), 3065–3071. 10.5897/ajmr12.239

[B22] Lee D. Y. Kim W. C. Kim J. G. (2012). Effect of Nitrite Concentration on the Corrosion Behaviour of Carbon Steel Pipelines in Synthetic Tap Water. Corrosion Sci. 64, 105–114. 10.1016/j.corsci.2012.07.005

[B23] Liang R. Grizzle R. S. Duncan K. E. McInerney M. J. Suflita J. M. (2014). Roles of Thermophilic Thiosulfate-Reducing Bacteria and Methanogenic Archaea in the Biocorrosion of Oil Pipelines. Front. Microbiol. 5 (89), 89–12. 10.3389/fmicb.2014.00089 24639674 PMC3944610

[B24] Little B. J. Blackwood D. J. Hinks J. Lauro F. M. Marsili E. Okamoto A. (2020). Microbially Influenced Corrosion-Any Progress? Corrosion Sci. 170, 108641. 10.1016/j.corsci.2020.108641

[B25] Liu B. Fan E. Jia J. Du C. Liu Z. Li X. (2021a). Corrosion Mechanism of Nitrate Reducing Bacteria on X80 Steel Correlated to its Intermediate Metabolite Nitrite. Construction Building Mater. 303, 124454. 10.1016/j.conbuildmat.2021.124454

[B26] Liu B. Sun M. Lu F. Du C. Li X. (2021b). Study of Biofilm-Influenced Corrosion on X80 Pipeline Steel by a Nitrate-Reducing Bacterium, *Bacillus Cereus*, in Artificial Beijing Soil. Colloids Surf. B: Biointerfaces 197, 111356. 10.1016/j.colsurfb.2020.111356 33007505

[B27] Liu H. Gu T. Asif M. Zhang G. Liu H. (2017). The Corrosion Behavior and Mechanism of Carbon Steel Induced by Extracellular Polymeric Substances of Iron-Oxidizing Bacteria. Corrosion Sci. 114, 102–111. 10.1016/j.corsci.2016.10.025

[B28] Machuca L. L. Lepkova K. Petroski A. (2017). Corrosion of Carbon Steel in the Presence of Oilfield deposit and Thiosulphate-Reducing Bacteria in CO 2 Environment. Corrosion Sci. 129, 16–25. 10.1016/j.corsci.2017.09.011

[B29] Magot M. Ravot G. Campaignolle X. Ollivier B. Patel B. K. C. Fardeau M.-L. (1997). *Dethiosulfovibrio Peptidovorans* Gen. nov., Sp. nov., a New Anaerobic, Slightly Halophilic, Thiosulfate-Reducing Bacterium from Corroding Offshore Oil wells. Int. J. Syst. Bacteriol. 47 (3), 818–824. 10.1099/00207713-47-3-818 9226912

[B30] Mansoori H. Brown B. Young D. Nešić S. Singer M. (2019). Effect of FexCayCO3 and CaCO3 Scales on the CO2 Corrosion of Mild Steel. Corrosion 75 (12), 1434–1449. 10.5006/3290

[B31] Marciales A. Peralta Y. Haile T. Crosby T. Wolodko J. (2019). Mechanistic Microbiologically Influenced Corrosion Modeling-A Review. Corrosion Sci. 146, 99–111. 10.1016/j.corsci.2018.10.004

[B32] Miller R. B. Lawson K. Sadek A. Monty C. N. Senko J. M. (2018). Uniform and Pitting Corrosion of Carbon Steel by *Shewanella Oneidensis* MR-1 under Nitrate-Reducing Conditions. Appl. Environ. Microbiol. 84 (12). 10.1128/AEM.00790-18 PMC5981061 29654179

[B33] Miranda D. A. Jaimes S. A. Bastidas J. M. (2013). Assessment of Carbon Steel Microbiologically Induced Corrosion by Electrical Impedance Spectroscopy. J. Solid State. Electrochem. 18 (2), 389–398. 10.1007/s10008-013-2262-5

[B34] Moura M. C. Pontual E. V. Paiva P. M. G. Coelho L. C. B. B. (2013). ““An Outline to Corrosive Bacteria,” in Microbial Pathogens and Strategies for Combating Them: Science, Technology and Education. Editor Méndez-Vilas A. (Badajoz: Formatex Research Center), 11–22.

[B35] NACE (2013). “SP0775 Preparation, Installation, Analysis, and Interpretation of Corrosion Coupons in Oilfield Operations,” in Standard Practice (Houston: Texas: NACE International).

[B36] Newman R. C. Wong W. P. Ezuber H. Garner A. (1989). Pitting of Stainless Steels by Thiosulfate Ions. Corrosion 45 (4), 282–287. 10.5006/1.3577855

[B37] Palacios P. A. Snoeyenbos-West O. Löscher C. R. Thamdrup B. Rotaru A.-E. (2019). Baltic Sea Methanogens Compete with Acetogens for Electrons from Metallic Iron. ISME J. 13 (12), 3011–3023. 10.1038/s41396-019-0490-0 31444483 PMC6864099

[B38] Ruebush S. S. Brantley S. L. Tien M. (2006). Reduction of Soluble and Insoluble Iron Forms by Membrane Fractions of *Shewanella Oneidensis* Grown under Aerobic and Anaerobic Conditions. Appl. Environ. Microbiol. 72 (4), 2925–2935. 10.1128/AEM.72.4.2925-2935.2006 16597999 PMC1449039

[B39] Salgar-Chaparro S. J. Castillo-Villamizar G. Poehlein A. Daniel R. Machuca L. L. (2020a). Complete Genome Sequence of *Shewanella Chilikensis* Strain DC57, Isolated from Corroded Seal Rings at a Floating Production System in Australia. Microbiol. Resour. Announc 9 (38). 10.1128/MRA.00584-20 PMC7498422 32943556

[B40] Salgar-Chaparro S. J. Darwin A. Kaksonen A. H. Machuca L. L. (2020b). Carbon Steel Corrosion by Bacteria from Failed Seal Rings at an Offshore Facility. Sci. Rep. 10, 12287. 10.1038/s41598-020-69292-5 32703991 PMC7378185

[B41] Scully J. R. (2000). Polarization Resistance Method for Determination of Instantaneous Corrosion Rates. Corrosion 56 (2), 199–218. 10.5006/1.3280536

[B42] Selim M. S. Shenashen M. A. El-Safty S. A. Higazy S. A. Selim M. M. Isago H. (2017). Recent Progress in marine Foul-Release Polymeric Nanocomposite Coatings. Prog. Mater. Sci. 87, 1–32. 10.1016/j.pmatsci.2017.02.001

[B43] Speight J. G. (2014). “Chapter e1-Corrosion,” in Oil and Gas Corrosion Prevention. Editor Speight J. G. (Boston: Gulf Professional Publishing), e1–e24. 10.1016/b978-0-12-800346-6.00001-6

[B44] Stott J. F. D. (2012). “Implementation of Nitrate Treatment for Reservoir Souring Control: Complexities and Pitfalls,” in SPE International Conference and Exhibition (Aberdeen, UK.

[B45] Wang D. Liu J. Jia R. Dou W. Kumseranee S. Punpruk S. (2020). Distinguishing Two Different Microbiologically Influenced Corrosion (MIC) Mechanisms Using an Electron Mediator and Hydrogen Evolution Detection. Corrosion Sci. 177, 108993. 10.1016/j.corsci.2020.108993

[B46] Wang Y. Cheng G. Li Y. (2016). Observation of the Pitting Corrosion and Uniform Corrosion for X80 Steel in 3.5wt.% NaCl Solutions Using *In-Situ* and 3-D Measuring Microscope. Corros Sci. 111, 508–517. 10.1016/j.corsci.2016.05.037

[B47] Weber K. A. Achenbach L. A. Coates J. D. (2006). Microorganisms Pumping Iron: Anaerobic Microbial Iron Oxidation and Reduction. Nat. Rev. Microbiol. 4 (10), 752–764. 10.1038/nrmicro1490 16980937

[B48] Xu D. Li Y. Song F. Gu T. (2013). Laboratory Investigation of Microbiologically Influenced Corrosion of C1018 Carbon Steel by Nitrate Reducing Bacterium *Bacillus Licheniformis* . Corrosion Sci. 77, 385–390. 10.1016/j.corsci.2013.07.044

[B49] Yuk S. Kamarisima A. H. Azam K. Tanji Y. (2020). The Contribution of Nitrate-Reducing Bacterium *Marinobacter* YB03 to Biological Souring and Microbiologically Influenced Corrosion of Carbon Steel. Biochem. Eng. J. 156, 107520. 10.1016/j.bej.2020.107520

[B50] Zhang W. Carcea A. G. Newman R. C. (2015). Pitting of Steam-Generator Tubing Alloys in Solutions Containing Thiosulfate and Sulfate or Chloride. Faraday Discuss. 180, 233–249. 10.1039/c5fd00008d 25898311

[B51] Zheng Y. (2015). Electrochemical Mechanism and Model of H_2_S Corrosion of Carbon Steel. Director of Dissertation: Srdjan Nesic: ProQuest Dissertations Publishing.

