# Supplementary material for: Corrosion of Carbon Steel by Shewanella chilikensis DC57 Under Thiosulphate and Nitrate Reducing Conditions
Source: Front Bioeng Biotechnol. 2022 Mar 10;10:825776. doi: 10.3389/fbioe.2022.825776 (PMC8961182; doi:10.3389/fbioe.2022.825776)
Supplement: Supplementary file 1 [file DataSheet1.pdf]

Supplementary material

Corrosion of Carbon Steel by *Shewanella chilikensis* DC57 Under Thiosulphate and Nitrate Reducing Conditions

Silvia J. Salgar-Chaparro[1], Johanna Tarazona[1], Laura L. Machuca[1*]

[1] Curtin Corrosion Centre, WA School of Mines: Minerals, Energy and Chemical Engineering, Curtin University, Kent Street, Bentley, WA 6102, Australia

*** Correspondence:**

Laura L. Machuca

l.machuca2@curtin.edu.au

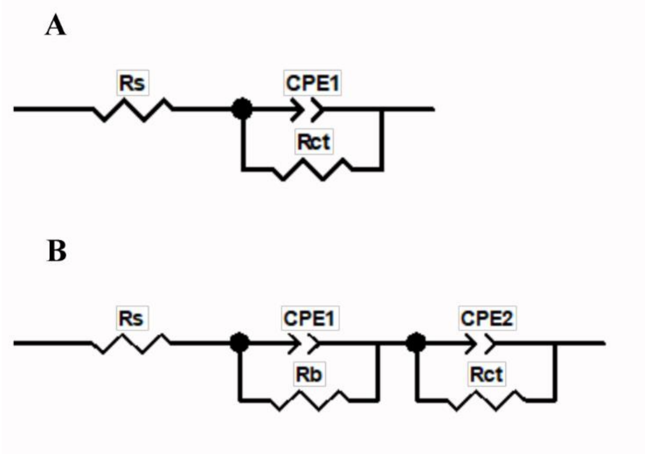

Figure S1. Equivalent circuit diagrams for EIS data fitting.

Table S1. *p*-value calculated from the t-test comparison of the pit depths.

Comparison	*p* value
Abiotic Vs Biotic<br>thiosulphate reducing conditions	0.0001
Abiotic Vs Biotic<br>nitrate reducing conditions	0.413
Biotic Vs Biotic<br>thiosulphate and nitrate reducing conditions	0.0001

Table S2. *p*-value calculated from the t-test comparison of the uniform corrosion rates.

Comparison	*p* value
Abiotic Vs Biotic<br>thiosulphate reducing conditions	0.014
Abiotic Vs Biotic<br>nitrate reducing conditions	0.028
Biotic Vs Biotic<br>thiosulphate and nitrate reducing conditions	0.028
